# Protocol for the Redefining Maternal Anemia in Pregnancy and Postpartum (ReMAPP) study: A multisite, international, population-based cohort study to establish global hemoglobin thresholds for maternal anemia

**DOI:** 10.1371/journal.pone.0321943

**Published:** 2025-07-28

**Authors:** Emily R. Smith, Zahra Hoodbhoy, Aneeta Hotwani, Fyezah Jehan, Amna Khan, Imran Nisar, Nida Yazdani, Santosh Joseph Benjamin, Anne George Cherian, Venkata Raghava Mohan, Sunitha Varghese, Balakrishnan Vijayalekshmi, Blair J. Wylie, Leena Chatterjee, Arjun Dang, R. Venketeshwar, Sasha G. Baumann, Christopher Mores, Qing Pan, Christopher R. Sudfeld, Victor Akelo, Winnie K. Mwebia, Kephas Otieno, Gregory Ouma, Harun Owuor, Joyce Were, Dennis Adu-Gyasi, Veronica Agyemang, Sam Newton, Charlotte Tawiah, Arun Singh Jadaun, Sarmila Mazumder, Neeraj Sharma, Lynda G. Ugwu, Amma Benneh-Akwasi Kuma, Bethany Freeman, Margaret P. Kasaro, Felistas M. Mbewe, Humphrey Mwape, Rachel S. Resop, M. Bridget Spelke, Kwaku Poku Asante, Redefining Maternal Anemia in Pregnancy and Postpartum (ReMAPP) Study Investigators

**Affiliations:** 1 Department of Global Health, George Washington University, Washington, DC, United States of America; 2 Department of Paediatrics and Child Health, Aga Khan University, Karachi, Pakistan; 3 Christian Medical College Vellore, Vellore, India; 4 Department of Obstetrics and Gynecology, Columbia University Medical Center, New York, New York, United States of America; 5 Dr. Dangs Lab, New Delhi, India; 6 Department of Global Health, Harvard T.H. Chan School of Public Health, Cambridge, Massachusetts, United States of America; 7 Kenya Medical Research Institute, Kisumu, Kenya; 8 Department of Clinical Sciences, Liverpool School of Tropical Medicine, Liverpool, England; 9 Kintampo Health Research Centre, Research and Development Division, Ghana Health Service, Kintampo North Municipality, Bono East Region, Ghana; 10 Society for Applied Studies, New Delhi, India; 11 Department of Maternal-Fetal Medicine, University of Alabama at Birmingham School of Medicine, Birmingham, Alabama, United States of America; 12 Department of Hematology, College of Health Sciences, University of Ghana, Accra, Ghana; 13 Department of Obstetrics and Gynecology, School of Medicine, University of North Carolina, Chapel Hill, North Carolina, United States of America; 14 University of North Carolina—Global Projects Zambia, Lusaka, Zambia; PLOS: Public Library of Science, UNITED KINGDOM OF GREAT BRITAIN AND NORTHERN IRELAND

## Abstract

**Background:**

Anemia affects one in three pregnant women worldwide, with the greatest burden in South Asia and sub-Saharan Africa. During pregnancy, anemia has been linked to an increased risk of adverse maternal and neonatal health outcomes. Despite widespread recognition that anemia can complicate pregnancy, critical gaps persist in our understanding of the specific causes of maternal anemia and the cutoffs used to diagnose anemia in each trimester and in the postpartum period.

**Methods and analysis:**

The Redefining Maternal Anemia in Pregnancy and Postpartum (ReMAPP) study is a multisite, prospective, cohort study nested within the Pregnancy Risk, Infant Surveillance, and Measurement Alliance (PRISMA) Maternal and Newborn Health study. Research sites are located in Kenya, Ghana, Zambia, India, and Pakistan. Participants are up to 12,000 pregnant women who provide serial venous blood samples for hemoglobin assessment at five time points: at <20 weeks, 20 weeks, 28 weeks, and 36 weeks gestation and at six weeks postpartum. We will use two analytical approaches to estimate hemoglobin thresholds for defining anemia: (1) clinical decision limits for cutoffs in each trimester and at six weeks postpartum based on associations of hemoglobin levels with adverse maternal, fetal, and neonatal health outcomes and (2) reference limits for gestational-week-specific cutoffs and at six weeks postpartum for mild, moderate, and severe anemia based on tail statistical percentiles of hemoglobin values in a reference (i.e., clinically healthy) subpopulation. We will also conduct biomarker-intensive testing among a sub-sample of participants in each trimester to explore underlying contributing factors of maternal anemia.

**Ethics and dissemination:**

The study received local and national ethical approvals from all participating institutions. Findings from multisite analyses will be published among open-access, peer-reviewed journals and disseminated with local, national, and international partners.

**Strengths and limitations:**

- Novel study design to allow multiple analytical approaches (clinical decision limits and reference limits) in the same population to establish hemoglobin thresholds.
- Use of gold standard methods and external quality assurance programs to ensure harmonized hemoglobin measurement across sites.
- Inclusion of biomarker-intensive study arm to examine the etiology of anemia among pregnant women.
- All data is contributed by populations historically underrepresented in research in low- and middle-income countries.

**Trials registration:**

ClinicalTrials.gov (PRISMA-MNH 2022; NCT05904145).

## Introduction

Anemia is a common health condition characterized by low blood hemoglobin concentration and/or low red blood cell count. Women, particularly those who are pregnant and lactating, are physiologically at a higher risk than men of being anemic [1,2]. An estimated 36% of pregnant women globally are anemic, with the highest prevalence in South Asia (48%) and West and Central Africa (52%) [3]. Although there are many causes of anemia, iron deficiency is estimated to account for roughly half of anemia cases in women [4]. Other contributing causes of anemia include infectious diseases (e.g., hookworm, malaria, schistosomiasis, HIV), gynecologic conditions (e.g., abnormal uterine bleeding, hemorrhage, uterine fibroids), other micronutrient deficiencies (e.g., folate, vitamin B12), thyroid dysfunction, chronic kidney disease, hemoglobinopathies like sickle cell disease or thalassemia, and environmental exposures such as lead or arsenic [1,2,5–8]. Importantly, causes of anemia vary widely by geography, life stage, and age.

During pregnancy, abnormal hemoglobin levels are linked to an increased risk of adverse maternal, fetal and neonatal health outcomes. Specifically, maternal anemia (i.e., low hemoglobin) can increase risk of postpartum hemorrhage, preeclampsia, maternal mortality, postpartum maternal depression, preterm delivery, low birth weight, small for gestational age, stillbirth, neonatal mortality, and poor infant brain structural development [9–13]. The likelihood of these outcomes depends on the case severity, gestational timing onset and duration of anemia [11,12]. Multiple studies have also observed an association between high hemoglobin concentration and pregnancy complications, though there are no standard thresholds to define excess hemoglobin [11,14–16]. In a systematic review and meta-analysis by Young et al. (2023), hemoglobin levels greater than 13.0 g/dL during pregnancy were associated with increased risk of small-for-gestational-age, stillbirth, (very) low birth weight, preterm birth, gestational diabetes, preeclampsia, and maternal mortality [17].

A growing body of evidence over the last decade suggests that the hemoglobin cutoffs used to diagnose anemia during pregnancy and the postpartum period should be reevaluated in consideration of data from low- and middle-income countries (LMICs) [11,15,18,19]. In 2017, a World Health Organization (WHO) technical consultation concluded that the current hemoglobin thresholds used in pregnancy were not supported by sufficient data, not proven to be linked to health outcomes, not representative of diverse geographies and ethnicities, and not necessarily applicable in the context of common gene mutations that affect hemoglobin function [20]. Although WHO released revised anemia definitions in 2024, the use of trimester-specific cutoffs during pregnancy and of non-pregnant adult cutoffs in the immediate postpartum period to diagnose anemia are not well-evidenced [21]. Similarly—given that causes of anemia are varied in severity, multifactorial, and regionally distinct—further research is needed to understand how anemia etiology differs for pregnant women, given that most research to date has focused on non-pregnant women [22].

Correct anemia identification carries significance not only for its prevention, diagnosis, and treatment at the individual level, but also for tracking progress toward global anemia reduction targets. Hemoglobin thresholds to diagnose anemia in pregnancy at all gestational ages were first established by the World Health Organization (WHO) in 1959 as <10.0 g/dL, then adapted in 1968 to <11.0 g/dL [23,24]. These definitions of anemia were based on a ‘normal range’ of hemoglobin values; wherein below the 2.5th percentile (-2 standard deviations (SDs) from the mean) is considered low hemoglobin (i.e., anemia) and above the 97.5th percentile (+2 SDs from the mean) is high hemoglobin. Data informing the ‘normal range’ was largely derived from four published studies in high-income countries (Norway, England, Wales, United States) with small sample sizes, not all of which included women [25–28]. In 1989, the Centers for Disease Control (CDC) set thresholds based on studies in Europe and the United States for each trimester: <11.0 g/dL in the first and third trimesters and <10.5 g/dL in the second trimester [29]. The WHO introduced thresholds for anemia severity in pregnant women in 2011, adopted the CDC trimester-specific thresholds in 2016, and added trimester-specific severity cutoffs in 2024 (Table 1) [21,30,31]. Notably, the WHO guidelines released in 2024 excluded data from LMICs in the pooled analysis that informed the update, citing that the unknown effect of infection and inflammation on hemoglobin coupled with their high prevalence in these settings precluded their inclusion [21].

**Table 1:** WHO (2024) thresholds for anemia for non-pregnant and pregnant women.

Population	Hemoglobin threshold (g/dL)			
	Any anemia	Mild anemia	Moderate anemia	Severe anemia
Non-pregnant women	<12.0	11.0–11.9	8.0–10.9	<8.0
Pregnant women	–	–	–	–
1st trimester (≤13 weeks)	<11.0	10.0–10.9	7.0–9.9	<7.0
2nd trimester (14–27 weeks)	<10.5	9.5–10.4	7.0–9.4	<7.0
3rd trimester (≥28 weeks)	<11.0	10.0–10.9	7.0–9.9	<7.0

We designed the Redefining Maternal Anemia in Pregnancy and Postpartum (ReMAPP) study to establish hemoglobin cutoffs using multiple analytical approaches for the diagnosis of anemia during pregnancy and within 42 days postpartum in LMIC populations. The specific aims are threefold: (1) to determine trimester-specific and postpartum hemoglobin thresholds for anemia diagnosis based on statistically significant increased risks of adverse maternal, fetal, and newborn health outcomes (i.e., decision limits); (2) to estimate gestational-week-specific and six-week postpartum hemoglobin thresholds for mild, moderate, and severe anemia diagnosis using the 5th and 2.5th statistical percentiles among a clinically healthy subpopulation (i.e., reference limits); (3) to describe the underlying contributing factors of anemia (i.e., etiology) in pregnancy in Ghana, Kenya, Zambia, Pakistan, and India.

## Materials and methods

### Study design

**Fig 1:**
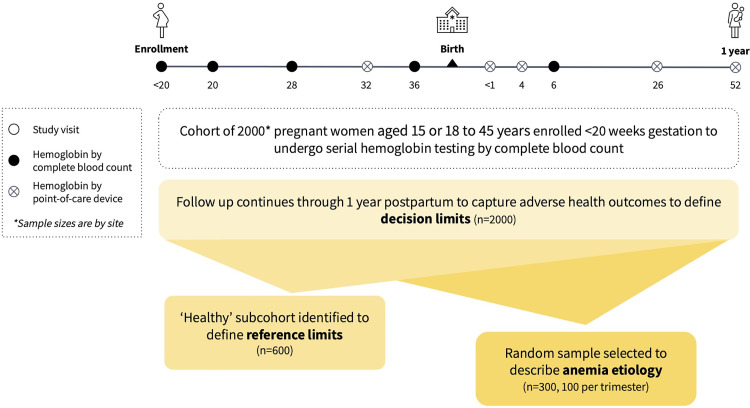
Nested ReMAPP study design with estimated 12,000 pregnancies in study cohort (n = 1,650 to 2,000 each site).

ReMAPP is a prospective cohort study in five countries. It is embedded within the Pregnancy Risk, Infant Surveillance, and Measurement Alliance (PRISMA) Maternal and Newborn Health study: a longitudinal, open cohort study that seeks to evaluate pregnancy risk and health and development for women and infants [32]. The population for ReMAPP is nested in the PRISMA study cohort. As illustrated in Fig 1, further selection will be done from the primary cohort of enrolled ReMAPP participants to identify a clinically ‘healthy’ analytical subcohort and cross-sectional sub-samples for etiology assessment. Data from the primary cohort, analytical subcohort, and cross-sectional sample will be used to achieve aims one, two, and three, respectively. Enrollment for ReMAPP was initiated on a rolling basis by site and began between September 2022 and December 2023 (22 September 2022 in Pakistan, 15 December 2022 in Zambia, 28 December 2022 in Ghana, 14 April 2023 in Kenya, 20 June 2023 in India (Vellore), and 12 December 2023 in India (Hodal). Enrollment is expected to be completed by April 2025, with all follow up and data collection concluding across sites by December 2025. Results will be disseminated by study aim; it is anticipated that all results will be shared by early 2026.

### Study setting

This study focuses on countries in South Asia and sub-Saharan Africa with high anemia prevalence [33]. There are six research sites: Kintampo, Ghana; Kisumu and Siaya, Kenya; Lusaka, Zambia; Karachi, Pakistan; Vellore, India; and Hodal, India. Each site has identified and mapped geographic catchment areas, which have at least 1500 deliveries per year.

### Eligibility criteria and recruitment

Participants eligible for the ReMAPP study are pregnant women who live within the catchment area, are <45 years and meet country-specific minimum age requirements (Ghana: 15 years of age; Kenya: 18 years of age or emancipated minors; Pakistan: 15 years of age or emancipated minors; Zambia: 15 years of age; India: 18 years of age), have a viable intrauterine pregnancy, are less than 20 weeks gestation as verified via ultrasound, intend to stay in the study area until six weeks post-delivery, and who provide informed consent.

### Participant selection and sampling

All participants enrolled in the PRISMA study will be invited to participate in the ReMAPP study if they meet eligibility criteria. Enrollment will continue until the target sample size (n = 1650 to 2000 per site) is achieved. These participants will form the ReMAPP primary cohort, and will undergo serial hemoglobin testing by complete blood count and be followed up at regular study visits through one year postpartum.

Upon enrollment, all ReMAPP participants will undergo screening based on predetermined criteria to obtain sociodemographic information, obstetric history, vital signs, anthropometric measurements, and laboratory results. If a participant meets the 21 screening criteria (Box 1), they will be included in the clinically ‘healthy’ analytical subcohort. Selected participants may also be excluded from the final ‘healthy’ analytical subcohort if any of the following occur during follow-up: multiple pregnancies previously not identified, severe conditions not initially evident including cancer, HIV, tuberculosis, or malaria, or severe pregnancy-related conditions requiring hospital admission including severe preeclampsia/eclampsia.

**Box 1. Screening criteria for clinically healthy subcohort**

1. Aged 18 to 34 years.
2. Gestational age at enrollment <14 weeks.
3. Pre-pregnancy or early pregnancy body mass index (BMI) of >18.5 and <30 kg/m^2^.
4. Mid-upper arm circumference >23 cm.
5. Height ≥150 cm.
6. Singleton pregnancy.
7. Systolic blood pressure <140 mmHg and diastolic blood pressure <90 mmHg.
8. No iron deficiency (serum ferritin >15 mcg/L adjusted for inflammation [34]).
9. No subclinical inflammation (CRP≤5 mg/L and/or AGP≤1 g/L).
10. No hemoglobinopathies (SS, SC, SE, EE, CC, SD-Punjab, Sβthal, Eβthal, Cβthal, CD-Punjab, ED-Punjab, D-D-Punjab, D-Punjabβthal, Thalassemia major, Thalassemia intermedia, or Alpha thalassemia).
11. Normal glucose-6-phosphate dehydrogenase (≥6.1 U/g Hb).
12. No reported previous low birth weight delivery.
13. No reported previous stillbirth.
14. No reported previous unplanned cesarean delivery.
15. No reported cigarette smoking, tobacco chewing, or betel nut use.
16. No reported alcohol consumption during pregnancy.
17. No current malaria infection (per rapid diagnostic test).
18. No current Hepatitis B virus infection (per rapid diagnostic test).
19. No current Hepatitis C virus infection (per rapid diagnostic test).
20. No known history or current chronic disease (cancer, kidney disease, or cardiac condition).
21. No known history or current HIV.

Among the participants screened, a cross-sectional sample of up to 300 participants (100 per trimester, both anemic and nonanemic) per site will be selected to undergo biomarker-intensive testing. Any method of population-representative sampling may be used within each trimester stratum, provided that selection occurs over the same time period to eliminate time bias.

### Follow-up procedures and data collection

Data will be collected using a harmonized set of forms developed for the PRISMA study [32]. Beyond the routine PRISMA study visit procedures, ReMAPP participants will provide biological specimens to undergo additional laboratory testing (Table 2). For hemoglobin, the primary measure of interest, participants will be serially assessed at less than 20 weeks, at 20 weeks, 28 weeks, and 36 weeks gestation and at six weeks postpartum by complete blood count using a venous blood sample and a five-part differential automated hematology analyzer. Additional testing will be done at enrollment, as a part of the screening laboratory tests for the clinically ‘healthy’ subcohort. Biomarker-intensive testing will be done for the sample of 300 participants (100 per trimester), either during their enrollment visit (gestational age less than 14 weeks), 20-week, or 32-week ANC visit. Methods and timing of laboratory assessments will be standardized across sites to ensure comparability and quality.

**Table 2:** Laboratory tests for the ReMAPP study.

Condition	Assessment	Method
**Serial hemoglobin tests at <20, 20, 28, and 36 weeks gestation and 6 weeks postpartum**		
Anemia	Hemoglobin*	Complete blood count using five-part differential hematology analyzer
**Screening tests at enrollment (<20 weeks gestation) to identify clinically healthy subcohort**		
Iron deficiency	Ferritin	ELISA^1^ (Quansys Q-plex^TM^ Human Micronutrient v2 (7-plex))
	Serum transferrin receptor	ELISA^1^ (Quansys Q-plex^TM^ Human Micronutrient v2 (7-plex))
Inflammation	C-reactive protein	ELISA^1^ (Quansys Q-plex^TM^ Human Micronutrient v2 (7-plex))
	Alpha 1-acid glycoprotein	ELISA^1^ (Quansys Q-plex^TM^ Human Micronutrient v2 (7-plex))
Communicable diseases	HIV	Rapid diagnostic test
	Malaria	Rapid diagnostic test
	Hepatitis B and C	Rapid diagnostic test
Red blood cell defects	Sickle cell disease	HPLC^2^
	Beta (β) and alpha (α) thalassemias	HPLC^2^
	Glucose-6-phosphate dehydrogenase	SD Biosensor Standard G6PD Analyzer
**Biomarker-intensive tests at <14, 20, or 32 weeks gestation for random sample**		
Iron deficiency	Hepcidin	ELISA^1^
	Total iron-binding capacity	Colorimetric chemistry analyzers
Other micronutrient deficiency	Vitamin A serum retinol	HPLC^2^
	Vitamin B12 total cobalamin	ECLIA^3^
	Vitamin B12 holotranscobalamin II	Abbexa HoloTC ELISA^1^
	Folate	Microbiologic assay
	Zinc	ICP-MS^4^ or AAS^5^
Red blood cell parameters	Red blood cell count	Five-part differential hematology analyzer
	Hematocrit	
	Mean corpuscular volume	
	Mean corpuscular hemoglobin concentration	
	Red cell distribution width	
	Red blood cell morphological abnormalities	Leishman, Wright-Giemsa, or Giemsa stain for microscopy
		White blood cell morphological abnormalities
		Platelet morphological abnormalities
		Presence of blood parasites
Communicable diseases	Syphilis	Rapid diagnostic test
	HIV	Rapid diagnostic test
	Hepatitis B and C	Rapid diagnostic test
	Malaria	Rapid diagnostic test
		Placental tissue histopathology
		Leishman, Giemsa stain, or Quantitative Buffy Coat for microscopy
	Tuberculosis	GeneXpert (4-module, 8-module, or 24-module)
	Schistosomiasis	Kato-Katz coproscopy, PCR^6^ test, or urine filtration
	Soil-transmitted helminths	Kato-Katz coproscopy
Non-communicable diseases	Urinalysis	Dipstick
	Renal function	Colorimetric chemistry analyzer
	Liver function	Colorimetric chemistry analyzer
	Blood lead	ICP-MS^4^ or AAS^5^

^1^Enzyme-linked immunoassay (ELISA).

^2^High-performance liquid chromatography (HPLC).

^3^Electrochemiluminescence (ECLIA).

^4^Inductively coupled plasma mass spectrometry (ICP-MS).

^5^Atomic Absorption Spectrophotometry (AAS).

^6^Polymerase chain reaction (PCR).

### Quality assurance for laboratory tests

Each site laboratory will adhere to the Laboratory Quality Assurance and Quality Control Standard Operating Procedures manual developed specifically for this study. For full blood count measurement, all participating labs will be enrolled onto the United Kingdom National External Quality Assurance Scheme for Hematology for monthly quality assurance [35]. For full blood count and all other analytes, the College of American Pathologists’ External Quality Assessment Program is required [36].

### Sample size estimates

Each site will enroll 1,650 to 2,000 participants in the ReMAPP study, to achieve an overall study sample of 12,000 participants across the six sites. The sample size was selected assuming that about 30% (n = 600) will meet the eligibility criteria for the ‘healthy’ subcohort. This sample size will also capture sufficient cases of the primary maternal and newborn outcomes of interest, based on previous regional prevalence estimates, in order to conduct a pooled decision limits analysis with at least 90% power to estimate association between anemia and each outcome.

**Fig 2:**
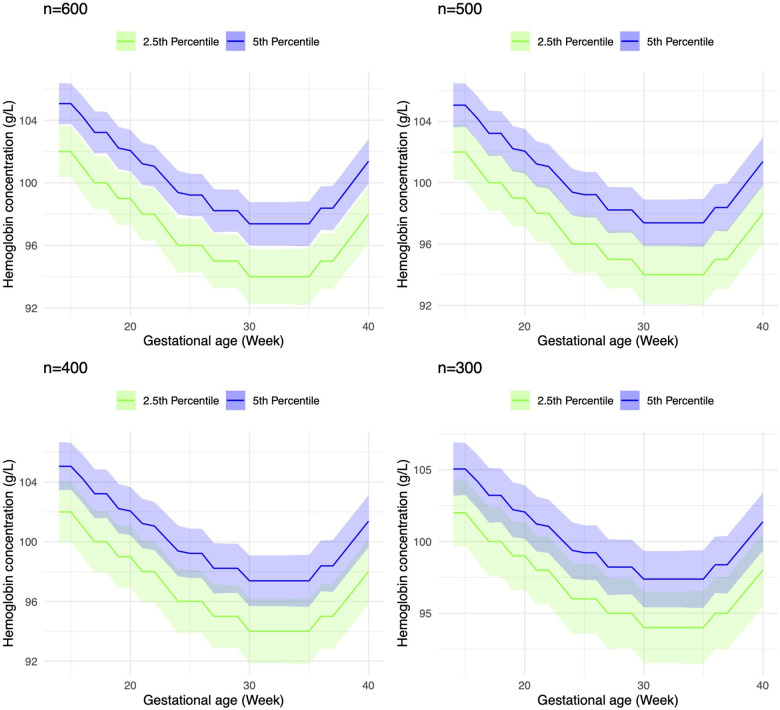
Visualization of sample size estimates with thresholds for any (i.e., 5th percentile) and severe maternal anemia (i.e., 2.5th percentile) at n = 300, 400, 500, 600.

Our power speculation is based on the distribution of hemoglobin values in pregnancy by gestational age presented in the INTERGROWTH-21st study [18]. For the ‘healthy’ analytical subcohort, a sample size of 600 participants per site allows for single-site estimation of hemoglobin thresholds for any maternal anemia (i.e., 5th percentile) and severe maternal anemia (i.e., 2.5th percentile). As illustrated in Fig 2, there will be sufficient precision to distinguish between these two percentiles without overlapping 95% confidence intervals. If sites are unable to identify 600 participants for the healthy cohort, a minimum sample size of 300 participants will be sufficient for single-site hemoglobin threshold estimation.

For the cross-sectional sample, n = 300 was determined to minimize the burden of additional specimen collection while allowing for exploratory pooled analyses of anemia etiology. The calculation is based on the following assumptions: one third of the participants have a certain contributing factor (e.g., the prevalence of vitamin A insufficiency); 30% of participants without the factor have anemia; and 50% with the factor have anemia. At the two-sided significance level of 95% and 80% statistical power, we can detect a prevalence ratio of 1.7 with a sample size of between 240 and 270 [37]. We inflated the sample to 300 to account for the fact that some contributing factors are less prevalent in certain study sites.

### Participant and public involvement

Participants will be collaboratively involved at multiple phases of the study. During initial protocol workshops, investigators carefully evaluated the additional burden of study participation; for this reason, the size of the biomarker-intensive sample was kept to a minimum and invasive blood draws limited to select visit timepoints. As a part of the translation and validation process for questionnaires to measure fatigue, patients’ experiences were centered and informed scale validation procedures. Participants’ health priorities and experiences will also be explored in corresponding qualitative research about maternal morbidity. Upon completion of the study, we intend to share the main findings with participants and community members via appropriate local dissemination methods. Finally, we will ensure that participants’ contributions to this research are acknowledged in any subsequent reports, presentations, and publications.

### Ethical and safety considerations

This protocol, the informed consent documents and any subsequent modifications will be reviewed and approved by the relevant institutional review board (IRB) and ethics review committee (ERC) responsible for oversight of the study at each site. IRB approvals for this research were received from the following ERCs in each country: Ghana (Ghana Health Service, GHC-ERC 019/09/20 and the Kintampo Health Research Center Institutional Ethics Committee, KHRCIEC/2020-17), Kenya (KEMRI Scientific and Ethics Review Unit, 04-10-358-4166), Zambia (University of Zambia Biomedical Research Ethics Committee, IRB00001131 of IORG0000774; University of North Carolina at Chapel Hill Biomedical IRB, Study #14-2113), India (Office of Research, Christian Medical College, Vellore, India Ethics Committee, IRB No. 14553; Ethics Review Committee, Society for Applied Studies, New Delhi, SAS/ERC/ReMAPP Study/2022, Department of Health Research, EC/NEW/INST/2022/DL/0140), Pakistan (National Institutes of Health—Health Research Institute, National Bioethics Committee Ref: No.4-87/NBC-962/23/593 and Aga Khan University Ethics Review Committee, 2022-7197-21350), and the United States (Columbia University IRB-AAAU7504; The George Washington University IRB NCR224396; Harvard University IRB23-1093; The University of Alabama at Birmingham IRB-300013081). The rights and safety of all study participants will be protected. Written informed consent, assent and parental consent will be sought from all study participants as appropriate prior to enrollment. Site-specific unique identification numbers will be issued to participants instead of names to protect their identity. A limited data set will be stored in a secure cloud server where only trained study staff will have credentials for access. Study participants with abnormal biochemical results will be referred for further clinical assessment and management according to study-specific standard operating procedures.

## Analytical plan

### Aim 1: Establishing hemoglobin clinical decision limits based on adverse outcomes

Aim 1 will establish trimester-specific and postpartum hemoglobin clinical decision limits for any anemia based on associations with adverse maternal, fetal, and infant outcomes (Table 3). For this analysis, hemoglobin and outcome data points from all participants will be pooled. Nonparametric relationships between risks of adverse events and hemoglobin levels will be plotted using real data [38]. A series of candidate thresholds in hemoglobin values will be considered and disease risks before and after each threshold will be compared. Thresholds with clinically meaningful differences in the disease risks and statistically sound significance levels will be selected. Clinical decision limits for overall adverse events will be reported, as well as disease-specific clinical decision limits.

**Table 3:** Primary and secondary health outcomes for establishing decision limits.

Outcome	Definition	Timing
**Primary outcomes**		
Severe maternal outcomes	Composite of maternal deaths, late maternal deaths, and near-misses	Pregnancy through 1 year postpartum
Maternal death	Death from any cause related to or aggravated by pregnancy or its management (excluding accidental or incidental causes)	Pregnancy through 42 days postpartum
Perinatal depression	Likelihood of depression per the Edinburgh Postnatal Depression Scale	At 20 weeks and 32 weeks gestation; at 6 weeks postpartum
Postpartum anemia	Low hemoglobin concentration <12 g/dL	6 weeks and 6 months
Preterm birth	Delivery prior <37 weeks of gestation of a live born infant	<37 weeks gestation
Small for gestational age^1^	Birthweight of lower than the sex-specific 10th percentile for each week of gestational age, according to the sex-specific INTERGROWTH reference standards	Delivery
Stillbirth	Delivery of a fetus showing no signs of life or>350g birth weight, if gestational age is unavailable	≥20 weeks gestation
**Secondary outcomes**		
Late maternal death	Death from any cause related to or aggravated by pregnancy or its management (excluding accidental or incidental causes)	From 42 days to 1 year postpartum
Preeclampsia	For participants without preexisting hypertension, defined as gestational hypertension AND gestational proteinuria OR development of severe features. For participants with chronic hypertension, defined as incident gestational proteinuria OR development of severe features	>20 weeks gestation to 42 days postpartum
Hemorrhage	Antepartum hemorrhage (APH): bleeding from or into the genital tract during pregnancy. Postpartum hemorrhage (PPH): estimated blood loss of ≥500 mL, clinical diagnosis of PPH, or receipt of a procedure to manage PPH (including blood transfusion).	≥24 weeks gestation and prior to delivery (APH), within 24 hours after birth (PPH)
Preterm premature rupture of membranes	Rupture of membranes before the onset of labor	<37 weeks of gestation
Maternal sepsis	Organ dysfunction resulting from infection	Pregnancy, delivery, and up to 42 days postpartum
Maternal fatigue^2^	Decreased capacity for physical and mental activity after childbirth, a persistent lack of energy, impairments in concentration and attention not easily relieved by rest or sleep	At 20 weeks and 32 weeks gestation; at 6 weeks postpartum
Low birthweight	Liveborn infant weighing less than 2500 g at birth	Within 72 hours of delivery
Neonatal hyperbilirubinemia	Presence of excess bilirubin. Calculated in line with the age-specific total bilirubin thresholds per the American Academy of Pediatrics BiliTool^TM^ algorithm [39]	Birth to 7 days
Possible severe bacterial infection	Presence of any of the following signs or symptoms: Not able to feed at all or not feeding well; convulsions; severe chest indrawing; high body temperature (≥38°C for <2 months, ≥37.5°C for 2+ months); low body temperature (less than 35.5°C); no movement/only when stimulated; fast breathing (≥60 BPM for <2 months, ≥50 BPM for 2+ months)	Birth to 59 days
Neonatal sepsis	Inflammatory response and organ dysfunction following presence of a severe infection from as suspected (by a clinician) or proven (with culture)	Birth to 28 days
Infant neurodevelopment^3^	Infant’s cognitive, motor, linguistic, and social-emotional development	At 12 months

^1^Small for gestational age defined as lower than the sex-specific 10th percentile for each week of gestational age per INTERGROWTH standards [40].

^2^Maternal fatigue assessed using the FACIT-Fatigue Scale at 20 weeks and 32 weeks gestation and six weeks postpartum.

^3^Infant neurodevelopment assessed using the Global Scale for Early Development Short Form.

### Aim 2: Establishing hemoglobin reference limits among a healthy population

Aim 2 will establish gestational-age-specific hemoglobin reference limits for any, mild, moderate, or severe anemia in a reference population (i.e., clinically ‘healthy’ subcohort) during pregnancy and within 42 days postpartum [41,42]. This analysis will include hemoglobin data points from all participants eligible for the clinically ‘healthy’ subcohort. Fractional polynomial regression (FPR) will be used to model and visualize the hemoglobin changing curves of different percentiles against gestational age [18]. Sensitivity analysis of the percentile curves predicted by FPR models will be performed excluding one site at a time. If the hemoglobin distributions are similar across sites and between-site between is less than within-site variance, we will pool the data; if not, analyses will remain stratified by site.

Hemoglobin levels below the 5th or 2.5th percentiles at the corresponding time points during pregnancy are typically considered abnormally low and likely indicative of anemia. Therefore, the reference limits are defined as tail percentiles of hemoglobin levels in the reference population. Tail percentiles, such as 5th or 2.5th percentiles, will be modeled as a smooth polynomial function of gestational weeks using hemoglobin levels. With the final validated model, we will estimate the 2.5th, 5th, 95th, and 97.5th percentiles predicted from the FPR curves at specific gestational timepoints from 14 to 40 weeks and postpartum.

### Aim 3: Describing the etiology of anemia in pregnancy

**Fig 3:**
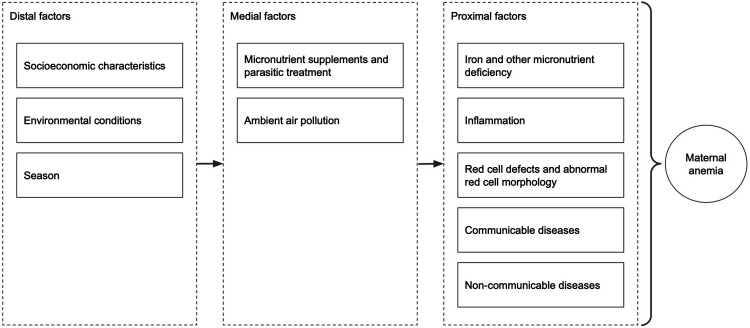
Risk factors of maternal anemia.

Aim 3 will describe the underlying contributing factors of anemia in pregnancy among a sample of up to 1,800 participants (up to 300 per site), with approximately equal numbers in each trimester. Analyses will be performed on pooled, regional, and site-specific data to identify shared and unique risk factors for anemia. We will quantify the association between contributing factors and anemia (defined using the WHO cutoffs and study-established reference and decision limit thresholds) by calculating relative risks from a generalized linear log-binomial model with robust standard errors. If these models fail to converge, we will use modified Poisson estimates, which produce valid but less efficient estimates of the log-binomial model. We will run separate models for proximal, medial, and distal risk factors (Fig 3). Distal risk factors will be included as potential confounders to proximal and medial models. We will estimate the population attributable risk fraction using prevalence of the exposure/risk factor and the relative risk of anemia in the exposed versus unexposed group. Partial population attributable risk will be calculated by fitting multivariate models with log link for anemia adjusting for multiple risk factors. For contributing factors significantly associated with risks of anemia, we will further report the change in continuous hemoglobin concentrations associated with the factor.

### Dissemination plan

The data generated in the course of the study will be reviewed on a regular basis for quality per our established Data Quality Assurance and Quality Control Standard Operating Procedures monthly. Following the end of the study, meetings will be organized at each study site to share results with community members, local authorities, and study participants. Findings will also be shared with local and international stakeholders, including the Bill & Melinda Gates Foundation, WHO, CDC, United States Agency for International Development, and Ministries of Health in each participating country. Abstracts will be developed for dissemination through both local and international scientific conferences and publication in peer-reviewed journals.
